# Assisted Grasping in Individuals with Tetraplegia: Improving Control through Residual Muscle Contraction and Movement

**DOI:** 10.3390/s19204532

**Published:** 2019-10-18

**Authors:** Lucas Fonseca, Wafa Tigra, Benjamin Navarro, David Guiraud, Charles Fattal, Antônio Bó, Emerson Fachin-Martins, Violaine Leynaert, Anthony Gélis, Christine Azevedo-Coste

**Affiliations:** 1 LARA, Department of Electrical Engineering, University of Brasília, Brasília 70919, Brazil; 2 INRIA, University of Montpellier, 34095 Montpellier, France; 3 MXM, 78153 Sophia Antipolis, France; 4 LIRMM, University of Montpellier, 34095 Montpellier, France; 5 CRF La Châtaigneraie, 95180 Menucourt, France; 6 Department of Physiotherapy at the Faculdade de Ceilândia, University of Brasília, Brasília 72220, Brazil; 7 PROPARA Clinical Center, 34090 Montpellier, France

**Keywords:** spinal cord injury, tetraplegia, FES-assisted grasping, inertial measurement unit interface, electromyography interface

## Abstract

Individuals who sustained a spinal cord injury often lose important motor skills, and cannot perform basic daily living activities. Several assistive technologies, including robotic assistance and functional electrical stimulation, have been developed to restore lost functions. However, designing reliable interfaces to control assistive devices for individuals with C4–C8 complete tetraplegia remains challenging. Although with limited grasping ability, they can often control upper arm movements via residual muscle contraction. In this article, we explore the feasibility of drawing upon these residual functions to pilot two devices, a robotic hand and an electrical stimulator. We studied two modalities, supra-lesional electromyography (EMG), and upper arm inertial sensors (IMU). We interpreted the muscle activity or arm movements of subjects with tetraplegia attempting to control the opening/closing of a robotic hand, and the extension/flexion of their own contralateral hand muscles activated by electrical stimulation. Two groups were recruited: eight subjects issued EMG-based commands; nine other subjects issued IMU-based commands. For each participant, we selected at least two muscles or gestures detectable by our algorithms. Despite little training, all participants could control the robot’s gestures or electrical stimulation of their own arm via muscle contraction or limb motion.

## 1. Introduction

Spinal cord Injury (SCI) may dramatically affect an individual’s ability to execute the activities of daily living. In a complete SCI, all the commands to muscles innervated by the segments of the spinal cord located from the injury down are interrupted. Cervical lesions result in tetraplegia, which induces the loss of lower limb motor skills, and causes complete or partial loss of upper limb control. Although lower limb disabilities result in a significant loss of overall mobility, not being able to perform routine manual tasks such as self-catheterizing, maintaining personal hygiene or feeding can have a devastating impact on the quality of life.

Nonetheless, although natural commands may no longer reach targeted muscles after a complete SCI, the muscles located below the lesion may have preserved motoneurons; this allows their activation via functional electrical stimulation (FES). FES can induce muscle contraction [1] and functional paralyzed limbs movements [2,3].

One major issue researchers face when working with FES—or other technologies for restoring human motor skills—is controlling movements through an interface capable of interpreting user commands.

Several non-invasive interfaces have been developed in the past, notably electroencephalography (EEG)-based brain computer interfaces (BCIs). Such devices aim at decoding brainwaves into user intentions. At this time, more work is needed to improve their accuracy and reliability [4,5]. Mechanomyography (MMG) has been used to read muscle vibrations, which can in turn serve to control devices by isometric muscle contraction [6]. However, this method is highly sensitive to limb movement artifacts, and therefore not reliable in real-life situations [7]. Electro-oculographic potential (i.e., tracking two-dimensional eye movements) has also been used to control a robotic arm [8]. Three-dimensional gaze-tracking would likely expand the control possibilities of this method, but its development remains challenging [9]. Voice commands have been used to control upper-extremity prostheses in silent environments [10]. Nevertheless, this method’s accuracy drops dramatically in noisy environments [11]. A common control method of choice is based on surface electromyography (EMG) signals [12]. In the past, several authors have proposed using surface EMG from the contralateral arm deltoid muscle to control a device which stimulated hand muscles [13,14]. The EMG signal from the ipsilateral wrist extensor muscles was used to control a hand neuroprosthesis. EMG signals have also been used to control an upper limb exoskeleton in [15]. Finally, body movement was used in [16] and [17] with camera-based systems and inertial measurement units (IMUs), respectively. In [18], contralateral shoulder motions were related to hand muscle stimulation by an external shoulder position transducer.

Non-invasive approaches remain more common: indeed, they require less complex technology and no surgery; however, they necessitate donning and doffing each time the system is used. Thus, invasive approaches to control arm and hand muscles with FES, such as head and neck-implanted EMG [19] or invasive BCIs [20,21], have recently been proposed.

At this developmental stage, movement or force-based sensors seem to offer the most realistic approach for user-intention recognition. However, these interfaces present challenges when motor skills are severely limited, such as in cervical-level SCI patients. Therefore, the use of these devices is usually limited to rehabilitation purposes [22].

In a preliminary study, we evaluated the ability of subjects with tetraplegia to contract supra-lesional muscles (trapezius, deltoid, platysma and biceps) as well as the comfort level in doing so, and assessed the feasibility of using EMG signals as an intuitive mode of controlling a robotic hand [23]. In this work, we investigate an easy-setup detection system considering further EMG control and exploring IMU-based control in the specific context of tetraplegia. Although the system was thought to be used in the future with a neuroprosthetic device, in the experiments presented in this work we validated the user interface capability to control the gestures of a robotic hand. Moreover, when possible, we applied the same control strategy to upper limb activation via FES of the contralateral hand muscles. Finally, we then assessed the ergonomics and performances of both modalities.

## 2. Materials and Methods

All participants gave their informed written consent to participate. The study was approved by the Ethical Committee (Comité de Protection des Personnes #2016-A00711-50, Sud Méditerranée IV, Montpellier, France) on 5 July 2016, and is in accordance with the 1975 Helsinki Declaration and its later amendments or comparable ethical standards. Two groups of participants with ASIA A or B tetraplegia were recruited from the Propara Neurological Rehabilitation Center in Montpellier, France, in two separate times. First, the EMG group was formed with 8 male subjects with lesions between C4 and C6 (Table 1). Later, for the it IMU group, another recruitment selected 9 male subjects with lesions between C4 and C7 (Table 2). One subject participated in both protocols and is identified as sE4 and sI5 in Table 1 and Table 2, respectively. The tables also indicate which individuals were able to use the FES system. All of them used the robotic hand.

### 2.1. Experimental Setup

**Figure 1:**
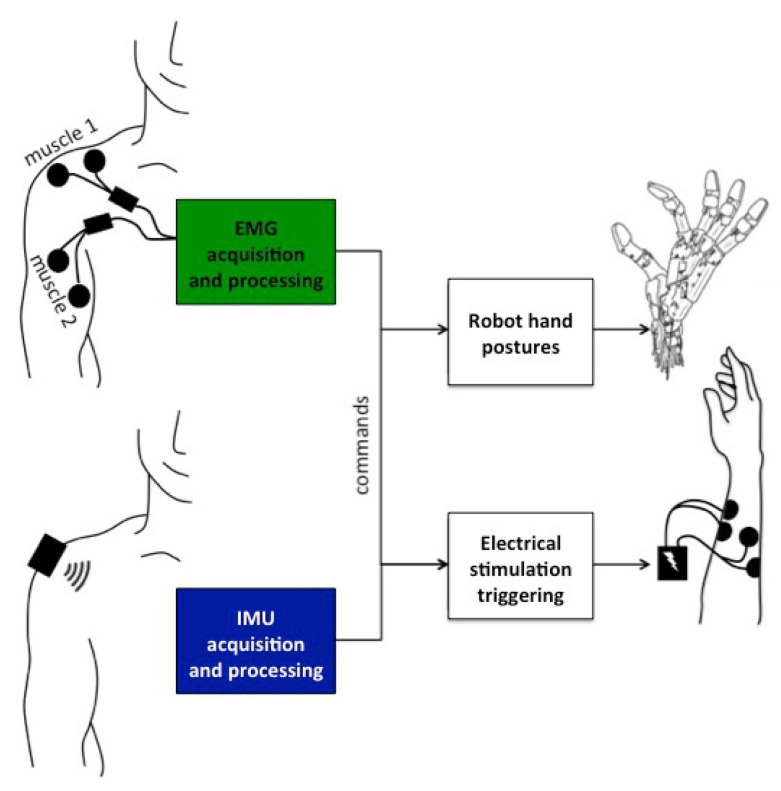
System diagram. The algorithms translate either EMG or IMU signals into commands for the robotic hand or the electrical stimulator. The robotic hand has three possible gestures: at-rest, open and close. The electrical stimulator can receive three commands: no stimulation, stimulate channel 1 (wrist flexion) or stimulate channel 2 (wrist extension). Users are able to observe the outcome of their input and use it as biofeedback.

Two sensing modalities and two actuation modalities were considered (Figure 1).

#### 2.1.1. *EMG Processing*

At the beginning of the session, 2 pairs of EMG surface electrodes were placed on different supra-lesional muscles: trapezius, biceps, platysma, deltoid posterior and deltoid anterior. Participants were asked to voluntarily contract those muscles for 4 seconds and the corresponding EMG signals were monitored. For each muscle, participants were asked to grade the level of comfort and easiness to perform a selective contraction. The muscle contraction EMG response was also analyzed to select 2 muscles that were finally used for the rest of the experiment and labeled: muscle 1 (M1) and muscle 2 (M2). The pair of muscles selected for the different participants are shown in Table 3.

**Figure 2:**
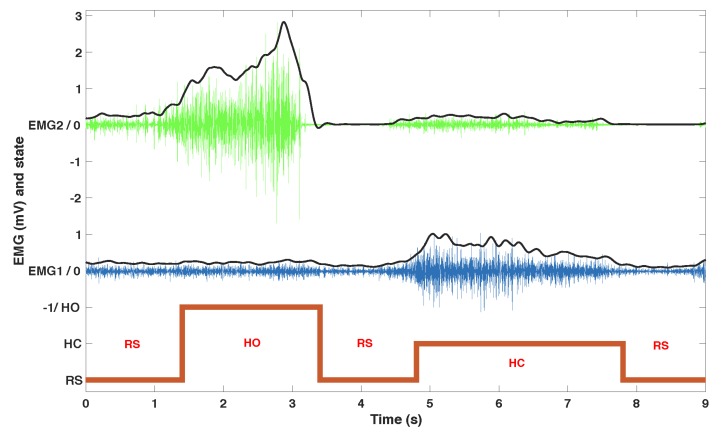
Representative example of EMG recordings with the automatic classification of states. Top: EMG 2. Middle: EMG 1. Bottom: actions (at-rest (RS), hand opening (HO) and hand closing (HC)). Black lines represent envelopes (their amplitude is multiplied by 4 for visualisation purposes).

The acquisition hardware consisted of an NI USB 6218, 16-bit, insulated acquisition board (National Instruments Corp., Austin, TX, USA), EMG was preconditionned by EMG amplifiers (Biopac Systems Inc., Goleta, CA, USA) with a gain set to 1000, and a battery powered laptop. The acquisition rate was 5 kHz to capture most of the EMG signal, then high-passed (20 Hz cutoff frequency 4th order Butterworth), rectified and then low-passed (4 Hz cutoff frequency, 4th order Butterworth) (Figure 2).

**Figure 3:**
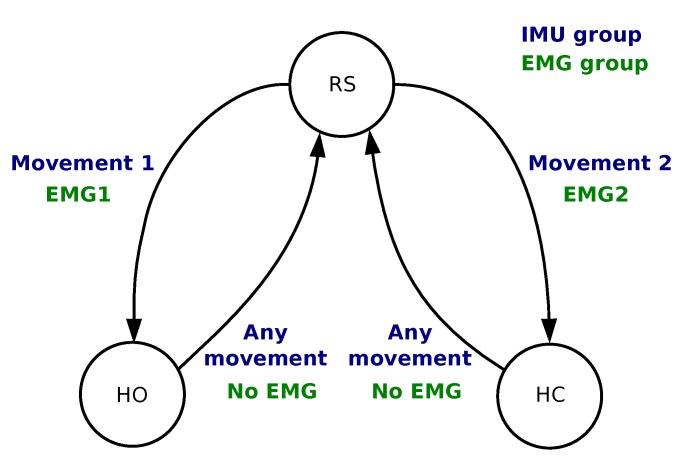
Finite state machine used to map 2 EMG or 2 movements to 3 commands: hand open (HO), hand close (HC) and rest state (RS).

We developed an algorithm to process EMG data in order to associate the muscle contractions with 3 predefined actions: at-rest (RS), hand opening (HO) and hand closing (HC). In the calibration phase, EMG data was recorded from each muscle at rest and with a strong voluntary contraction (VC) maintained for 4 s. The subject was instructed to increase the contraction if it was not clearly visible on the EMG signal. Four thresholds were set as fixed ratios of the normalized EMG envelope against VC ($EMG_{Env}^{1}$, $EMG_{Env}^{2}$): two ratios per muscle ($RL_{m}$, $RH_{m}$) in order to provide hysteresis and command stability. The ratios were set for each patient in two phases during a learning session which lasted approximately 10 min. The first phase set two ratios for each muscle as 0.2 and 0.6 times the VC: It guarantees a robust detection of a single muscle contraction that can directly be used to control a single action. However, some patients felt discomfort from the contraction which would lead to manual tuning of the thresholds to either limit the required strength (higher threshold) or the level over which it should be maintained (lower threshold). On the second phase we empirically fine tuned these ratios in a customized configuration for each participant while using both muscles to manage possible co-contractions and further adjusting the easiness of the control based on feedback from the participants. The commands were triggered based on the finite-state machine depicted in Figure 3. The 4 conditions were as follows:

- *From RS to HC* Condition is TRUE if
  $EMG_{Env}^{1}>RH_{1}$ AND $EMG_{Env}^{2}<RL_{2}$;
- *From RS to HO* Condition is TRUE if
  $EMG_{Env}^{2}>RH_{2}$ AND $EMG_{Env}^{1}<RL_{1}$;
- *From HO/HC to RS* Condition is TRUE if
  $EMG_{Env}^{1}<RL_{1}$ AND $EMG_{Env}^{2}<RL_{2}$.

The algorithm was implemented in Matlab (Natick, MA, USA).

#### 2.1.2. *IMU Processing*

In order to capture the participants’ shoulder or arm movement, we used a wireless inertial measurement unit (Hikob, Meylan, France), with a sample frequency of roughly 47 Hz. Although this unit provides 3-axis accelerometer, gyroscope and magnetometer data (including processed Euler angles), we only used the accelerometer and gyroscope data. We avoided using the magnetometer because calibration and sensitivity may vary with the environment—a potential limitation, especially in an implanted version of the solution. The IMU was placed on different locations of the shoulder or upper arm, depending on the participants’ ability to perform repeatable movements. As long as the IMU is well attached to the user throughout the experiment, any positioning on the participant moving body part would work, e.g., on the lateral part of the shoulder if the participant is performing a shoulder movement, or on the wrist if they are performing forearm movements. We developed an algorithm to process IMU data in order to associate 2 movements (movement 1 and movement 2) with the same predefined commands as in Section 2.1.1: RS, HC and HO. For that purpose, we used a finite-state machine as shown in Figure 3.

In the calibration phase, we asked the subjects to perform various movements with both shoulders. We typically instructed them to move the shoulder up, forward and backward, always returning to the initial position. These movements should be performed quickly, with a rest period in the initial position between them. If they had any difficulties doing so, if they felt tired, or if their movements seemed too slow (more than 1 s) or not very consistent (every repetition looking different), we asked them to move their upper arm forward, backward and outward. If the results still seemed inadequate, we asked them to move their forearm upward and inward. We requested that each movement be executed multiple times for 10 s, with 1 s intervals between each repetition. That procedure was carried out for each different calibrated movement in order to acquire reference signals. The data were differentiated, thereby providing a vector *X*, where each element $X_{i}$ refers to one axis of an individual sensor (i.e., accelerometer or gyroscope), and resulting in six elements. Then, thresholds were calculated using:

(1) $$\alpha_{i}=\frac{max(X_{i})}{2}$$

We used α to find movements performed during the initial calibration phase. At any instant *k*, whenever $X_{i,k}>\alpha_{i}$, 1≤i≤6, we calculated a feature vector θ by extracting the root mean square for the last second of signal acquisition:

(2) $$\theta_{i}=\sqrt{\frac{1}{N}\sum_{j=k-N/2}^{k+N/2}X_{i,j}^{2}},$$

where N=1/f, and *f* is the average signal frequency during the last second. *f* was calculated at every step because the wireless IMU sample frequency occasionally dropped. Finally, θ represents a new movement. This calculation was performed offline, after the calibration movements were completed.

We carried out principal component analysis (PCA) on all movements, which were treated as six dimensions points, corresponding to three accelerometer axes and three gyroscope axes. The two principal components were considered so that all movements were reduced to two dimensional points. The centroids for each class of movement were calculated as the average coordinates of all points in each class.

**Figure 4:**
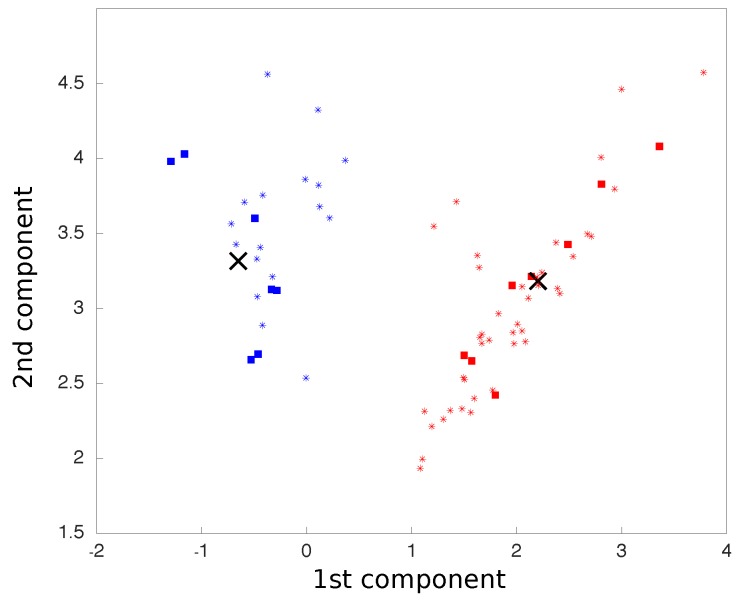
Representative example of movement classification with the IMU. Squares represent the movements used for calibration whereas stars are the movements classified online. The big “X” are the classes centroids.

After this calibration phase, we used the aforementioned process to find a new 2D point for each new movement, now using the pre-calculated PCA matrix, as in:

(3) $$M=P\cdot \theta_{new},$$

where *M* is a vector representing the new point coordinates on the PCA space, calculated by the cross product between $\theta_{new}$, the new point six-dimensional representation vector before the PCA rotation, and *P*, the PCA matrix previously calculated. The two-dimensional plot representing the two main components of the PCA was updated in real time as each movement was processed (Figure 4).

The new point distance to each centroid was calculated and, based on the shortest Euclidean distance, classified and associated with a finite-state machine action (Figure 3). All movements were classified into one of the two classes. The following conditions were implemented:

- *From RS to HO* Condition is TRUE if
  Movement ∈ class 1;
- *From RS to HC* Condition is TRUE if
  Movement ∈ class 2;
- *From HO/HC to RS* Condition is TRUE if
  Movement ∈ class 1 OR Movement ∈ class 2.

The algorithm was implemented in Matlab (Natick, MA, USA).

#### 2.1.3. *Actuation Modalities*

##### Robotic Hand

**Figure 5:**
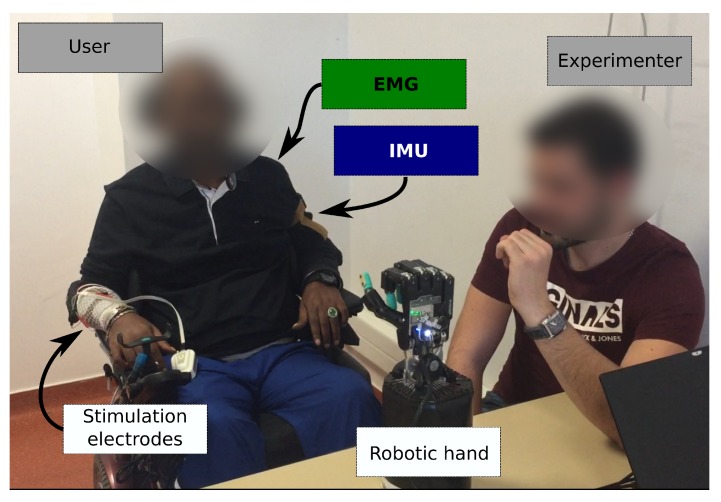
Setup for EMG and IMU sessions. In the validation phase, an experimenter showed the subject which gesture the robotic hand should be commanded to execute.

We chose to use a robotic hand for validation, training and feedback. Thus, users were able to monitor the outcome of their movements and the algorithm activation on the robotic hand responses. It represented a physical moving object, possibly more relatable than a virtual representation or other simpler feedback interface. We used the Shadow Dexterous Hand (Shadow Robot Company, London, UK) and configured three different hand gestures: the *at-rest (RS)* position featured a natural hand at-rest position; the *opened (HO)* position consisted in having all fingers fully extended; and the *closed (HC)* position consisted in flexing the fingers into a key grip position. The robotic hand was placed in front of the users so they could see it and observe its response to their commands. This placement is shown in Figure 5.

##### Electrical Stimulation

We used a wireless electrical stimulator (Phenix© Neo USB, Montpellier, France) to activate the subjects’ forearm muscles. We used 2 channels to induce hand flexion and extension. Since our aim was not to study functional grasping, we optimized the resulting movement so as to provide visual feedback for the participant. We thus placed electrodes and set stimulation parameters to obtain a contraction sufficient to elicit a clearly visible movement of the fingers or the wrist. We used auto-adhesive 5 × 5 cm surface electrodes and set the stimulation parameters as follows: frequency, 25 Hz; pulse width, 300 μs current intensity was adjusted for each muscle and for each subject. The waveform was rectangular, biphasic and balanced.

### 2.2. Experimental Protocol

Each modality consisted of one session per subject. At the beginning of each session, we first assessed the possibility of activating wrist or finger flexors and extensors with FES. If possible, that participant would be included in both the robotic hand and the FES actuation modality protocols. But sometimes a response could not be elicited with FES, usually because the muscles were denervated or too weak. If that was the case, they would take part only in the robotic hand protocol. We then equipped the opposite arm with the sensors according to the desired modality and group (EMG or IMU). We chose not to position the sensors on the ipsilateral arm to avoid the resultant FES induced muscle contraction or movement from interfering with the participant volitional input.

A preliminary calibration phase was performed as described in the EMG and IMU processing Section 2.1.1 and Section 2.1.2.

Once the calibration procedure was completed, participants were encouraged to test the system for a period of 5 min by contracting the two muscles independently (EMG group) or performing the two movements (IMU group), and to observe the induced robotic hand’s open, close and at-rest gestures.

This training phase was followed by the validation phase. An experimenter sat in front of the participant and moved his own hand to indicate to the participant which hand gesture to execute on the robotic hand (Figure 5). The sequence was generated randomly and included 5 transitions from RS to HO, 5 transitions from RS to HC, 5 transitions from HO to RS and 5 transitions from HC to RS.

This exercise tested both the subject’s ability to correctly choose the required action to activate the desired robotic hand gesture and the overall system’s ability to correctly identify the subject’s action. Performance was measured by comparing how many indicated hand postures the participants were able to correctly activate.

At the end of the trial, the participants answered a questionnaire to evaluate effort, fatigue and comfort in completing the exercise, as well as their perception of the device’s overall operation.

The entire robotic hand procedure was repeated for the subjects who participated in the FES actuation modality session of the protocol.

## 3. Results

### 3.1. EMG Modality

All EMG group participants were able to complete the task of independently contracting the two selected muscles in order to pilot the finite-state machine.

**Figure 6:**
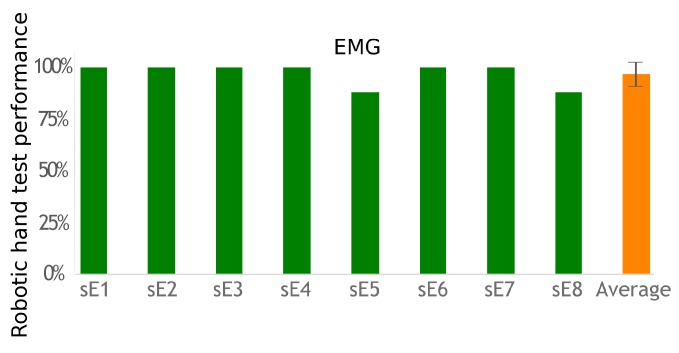
Performance results with the EMG system.

Figure 6 shows the performance achieved with the system and assessed in the robotic hand modality. Performance was defined as the number of correctly activated robotic hand gestures divided by the number of desired gestures. The average outcome was 97%±6%, and six out of eight participants achieved 100%. The participants involved in the FES modality tests were also able to pilot their hand opening/closing. However, their performance could not be measured in a standardized manner since stimulation sites and activated muscles were too diverse between them.

### 3.2. IMU Modality

**Figure 7:**
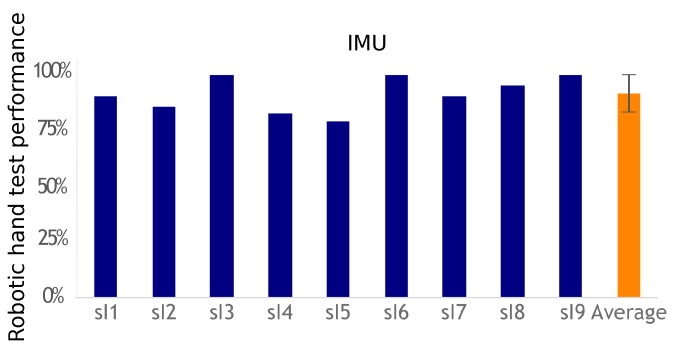
Performance results with the IMU system.

All IMU group participants were able to complete the task of controlling two movements which were classified and associated with specific actions. Figure 7 shows the performance achieved with this system and assessed in the robotic hand modality. The average outcome was 91%±8%. Moreover, three out of nine participants achieved 100% performance.

Furthermore, the IMU algorithm had no false negatives or positives. The threshold automatic calculation turned out to be quite robust. All errors were due to wrong movement classification.

Similar to the EMG modality, the participants involved in the FES actuation modality tests were also able to pilot their hand opening/closing, but their performance could not be measured.

### 3.3. Questionnaire

**Figure 8:**
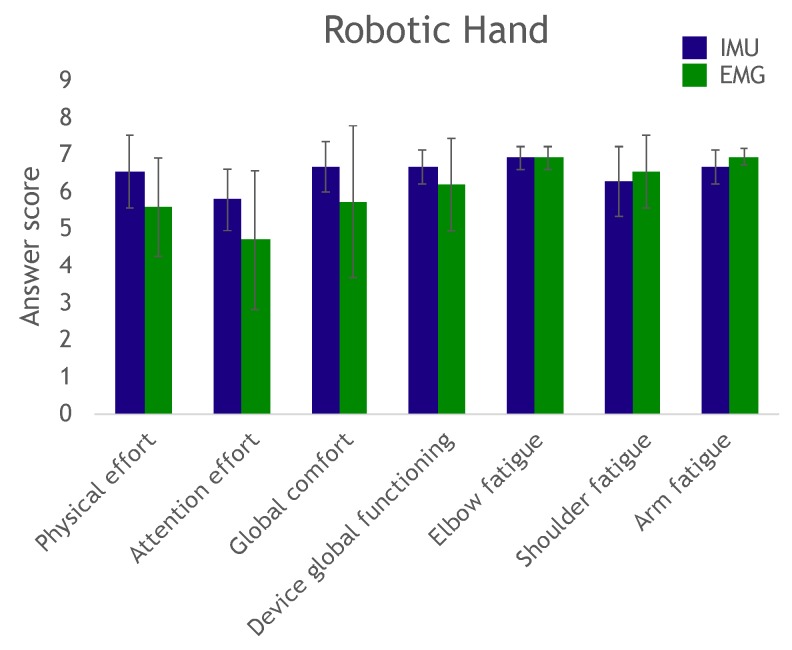
Questionnaire results for the robotic hand control task. The higher the value, the more positive the subject’s perception. The maximum score is always 7.

**Figure 9:**
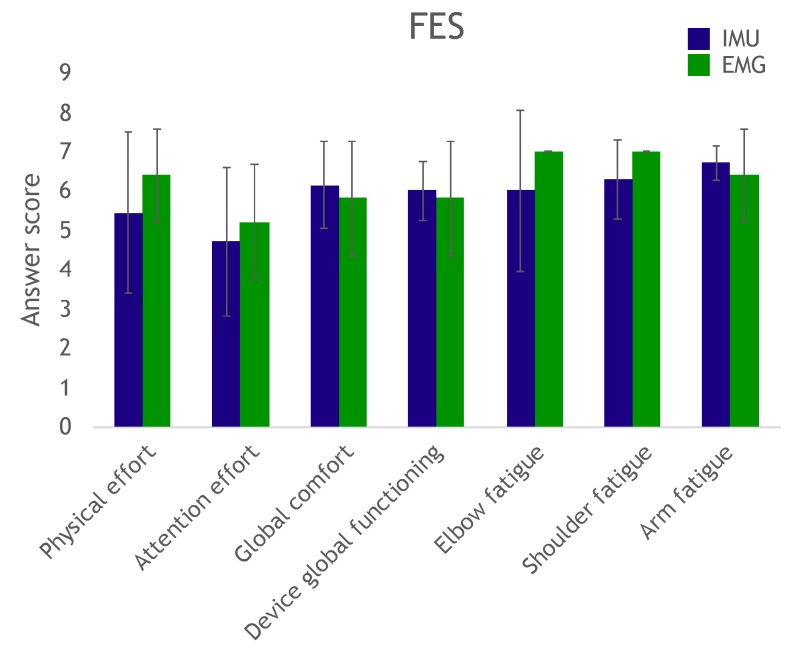
Questionnaire results for the functional electrical stimulation (FES) control task. The higher the value, the more positive or easier the subject’s perception. The maximum score is always 7.

An oral questionnaire was administered to evaluate participant perception of required effort (physical, attention), fatigue and overall comfort. A numeric scale from 1 to 7 was provided; the higher the number, the higher the perception of the system’s ease of operation. Responses were averaged and are shown in Figure 8 and Figure 9. We deemed scores above 6 as satisfactory, which was the case for arm, shoulder and elbow fatigue in both groups, and in the various modalities and actuations (average: 6.62±0.33). Likewise, overall system operation (average: 6.15±0.35) and overall comfort (average: 6.07±0.42) also had good scores. The required physical effort score was borderline (average: 5.97±0.56). Finally, the required attention effort had a lower score (average: 5.08±0.51), implying the system required a certain level of attention from the user.

### 3.4. Other Observations

Whenever FES was used, users were able to control their own hand with the system’s assistance. In several participants, we found it somewhat difficult to identify the motor points upon which to place the electrodes which would enable them to perform grasping movements. However, in most cases, we were able to activate wrist extension or lower arm rotation muscles. Several subjects had never experienced FES before. They were often in awe of their own limbs’ movements. This would sometimes distract them from the visual or auditory cues (e.g., movements to perform) given by the experimenters.

During the development phase, our subjects stressed the importance of low system latency, requesting that the delay between their muscle contraction or limb movement and the reaction of the robotic hand or the electrical stimulation be as short as possible. For instance, in the IMU group, the RMS was calculated at the end of a 1 s window around each movement. Since the movement was in the center of this time window, the system’s response delay was approximately 0.5 s. One of the subjects reported that this delay still felt too long.

## 4. Discussion

We investigated the capability of individuals with tetraplegia to control either a robotic hand or their own hand through two modalities. The objective was to assess whether the supra-lesional muscles could be voluntarily controlled in a sufficiently repeatable and selective manner to be used as piloting orders.

The EMG-based system allows for the easy tuning of thresholds; nevertheless, several issues need to be considered. Indeed, tuning consists in achieving a delicate balance between providing subjects with a very sensitive command so that even non-functional weak muscles can be used, and limiting the influence of undesired co-contractions of the second muscle. Meeting these two goals implies striking a compromise between low and high thresholds. Future developments may focus on a cross learning method between users that learn to properly activate their muscles and automatic thresholds setting based on overall performance. In our work, however, once the thresholds were set, within only a few minutes of training users were able to operate the system efficiently with little cognitive effort (assessed by the questionnaire). We chose to use continuous—versus pulse—muscle contraction command [23]. Though continuous contraction can induce more fatigue, the relationship between the contraction and the command is more direct.

EMG-based control requires equipping one muscle for each action to control, whereas IMU-based control requires only one sensor to control the transitions between actions. Using a unique sensor requires a classification method to identify the various movements associated with the participant’s commands. The classifier has to be trained. The calibration phase is more complex than the EMG threshold detection solution. However, placing a unique IMU with no need for accurate placement is easier than placing two pairs of EMG electrodes. This work did not evaluate day-to-day performance and practicality, but, in both cases, electrode or sensor positioning is important to achieve similar results. However, the calibration process would take no more than 2 min for a trained subject used to operate the system, which would not have a big impact on its usability once the sensors are set up. Regardless of the modality, however, a helper would still be necessary as users often lack the ability to don and doff the equipment by themselves. Nevertheless, both approaches were successful (more than a 90% success rate), with no statistical difference between their performances. Improving individual fit can be achieved, but custom-fitting depends on individual residual capabilities for movements or muscle contractions. Furthermore, future works shall test multiple uses of the system across different days with a single calibration and classifier training. This would evaluate these processes robustness.

The IMU method studied here successfully classified two movements. These movements can be mapped as desired to perform different tasks on a assistive device, and also be expanded to more than two or three tasks if sequences of movements are considered. Also, it can be improved to classify more movements, as in [24]. Furthermore, a “do nothing” command should be associated with movements that are not recognized as one of the calibrated movements instead of “forcing” the movement into one of the predefined classes as done in this study.

One issue with the EMG solution is the possible presence of co-contraction of the two selected muscles, which may confuse the decision algorithm. In the future, a classifier could improve the robustness of the EMG approach by allowing the co-contraction of several muscles as a user command and taking advantage of muscle synergies occurring within the execution of a movement.

Furthermore, combining and consolidating both EMG and IMU information could be considered [25]. One possible solution was recently proposed by [26], in which proportional control is provided by an IMU on a headset, while discrete controls are activated by EMG signals from upper limb muscles. In that work, wireless custom made sensors included both IMU and EMG modalities.

It should be noted that, for both modalities to be implemented outside the laboratory, the interface should be switched off or the movements/contractions filtered so as not to unintentionally activate the neuroprosthesis.

The two control interfaces proposed in this work could potentially become a practical neuroprosthesis user interface for both rehabilitation and daily use. Most current commercially available devices for grasping restoration rely on buttons for operation [27]. They are restricted to rehabilitation context as there is the need of a therapist or helper to configure and activate the system. This is not the case in our solution, which users can control according to their will. Our system is also simpler than invasive devices that decode signals directly from the brain [21,28].

In our study, the visual feedback provided by the robotic hand or, to a lesser extent, by the subject’s own hand, was valuable to them; however, they could only see if the commands were in fact the desired ones or not. More precise biofeedback could therefore be provided to further enhance user performances. With the EMG-based control interface, the signal envelope and the thresholds are visually meaningful; with the IMU, movement distance to the class centroids can be visualized to check for movement reliability and repeatability.

The questionnaire results showed that several subjects reported scores below 4 in the required attention effort to control the device: These subjects needed to remain deeply concentrated on the task. We believe this was mostly due to the fact that they did not have any prior training. Remotely controlling a robot can be a highly unusual experience, and even more so when attempting to move a paralyzed hand. These subjects would have most likely been able to complete the task more confidently and with more ease after a couple of days of training (as did several other subjects with just minutes of practice).

In addition, we assume the overall performance would also increase with further training. Extensive use of the system would likely make both muscle contractions and movements interfaces more precise and less demanding. With practice, participants should improve their muscle control which would enable them to activate the neuroprosthesis with lower effort, requiring new EMG thresholds. The same effect would be expected in the IMU modality. As users improve their movement precision and control, the classifier most likely would have to be retrained. Another possible consequence would be less fatigue from using the system, both from less demanding contractions and movements but also from possible muscle development, particularly those that are usually not used much due to lack of grasping function.

Our proposed finite-state machine control process implies that users can activate predefined prosthesis actions such as closed hand or open hand. Therefore, it does not allow any force control, or any other continuous control. It would be easy, in the EMG approach, to relate muscle contraction intensity to movement amplitude or grasping force intensity [23]. Likewise, with training, users are expected to develop precise movements, which would enable the classifier to learn a larger number of movements.

All these possibilities would thus allow researchers to customize solutions to users depending on their individual capabilities and limitations. However, the few number of commands and the necessity to don and doff the sensors and electrodes every time are meaningful limitations. Therefore, besides combining both EMG and IMU solutions in one single system, future works should include the implementation of more commands with both discrete triggering and proportional control, implantation of some systems parts such as sensors and electrodes and experiments that last for several days with the same equipment. These will advance the technology towards practical use.

## 5. Conclusions

Even without significant prior training, low-level tetraplegic subjects were able to accurately harness both residual muscular activity and shoulder/arm movements to control the actions of a robotic hand or of their own upper limb with an electrical stimulator. The most appropriate sensing modalities can be different for each user, and must therefore be set according to specific individual skills and residual motor skills. Considering that these individuals are highly dependent upon others to perform basic daily living activities, this result is promising. Future work involving advanced electrical stimulation approaches and combined sensors strategies should thus lead to significant improvements for the quality of life of individuals with tetraplegia.

## Figures and Tables

**Table 1:** Subject characteristics for the electromyography (EMG) group.

Subject	Sex	Age	Lesion Level	Lesion Time	FES
sE1	M	26	C6-AIS B	2 years	Yes
sE2	M	45	C5-AIS A	3 years	No
sE3	M	39	C5-AIS A	4 years	Yes
sE4	M	56	C5-AIS A	3 years	Yes
sE5	M	33	C4-AIS A	6 years	No
sE6	M	52	C6-AIS A	24 years	Yes
sE7	M	26	C6-AIS B	2 years	Yes
sE8	M	55	C5-AIS A	1 year	Yes

**Table 2:** Subject characteristics for the inertial sensors (IMU) group.

Subject	Sex	Age	Lesion Level	Lesion Time	FES
sI1	M	25	C6-AIS B	3 years	Yes
sI2	M	63	C7-AIS A	34 years	Yes
sI3	M	44	C5-AIS A	<1 year	Yes
sI4	M	40	C5-AIS A	3.5 years	No
sI5	M	56	C5-AIS A	3 years	Yes
sI6	M	51	C4-AIS A	33 years	Yes
sI7	M	65	C7-AIS B	47 years	No
sI8	M	25	C6-AIS A	3 years	Yes
sI9	M	19	C5-AIS B	<1 year	Yes

**Table 3:** Muscles chosen in EMG group. Muscle 1 (M1) contractions are associated with hand opening and muscle 2(M2) contractions are associated with hand closing.

Subject	M1	M2
sE1	trapezius sup	platysma
sE2	biceps	trapezius sup
sE3	biceps	deltoid post
sE4	deltoid post	biceps
sE5	biceps	trapezius sup
sE6	deltoid ant	biceps
sE7	biceps	deltoid post
sE8	biceps	trapezius sup
